# A Low-Prevalence Single-Nucleotide Polymorphism in the Sensor Kinase PhoR in *Mycobacterium tuberculosis* Suppresses Its Autophosphatase Activity and Reduces Pathogenic Fitness: Implications in Evolutionary Selection

**DOI:** 10.3389/fmicb.2021.724482

**Published:** 2021-08-25

**Authors:** Uchenna Watson Waturuocha, M. S. Krishna, Vandana Malhotra, Narendra M. Dixit, Deepak Kumar Saini

**Affiliations:** ^1^Department of Studies in Zoology, University of Mysore, Mysore, India; ^2^Department of Molecular Reproduction Development and Genetics, Indian Institute of Science, Bengaluru, India; ^3^Sri Venkateswara College, University of Delhi, New Delhi, India; ^4^Department of Chemical Engineering, Indian Institute of Science, Bengaluru, India; ^5^Center for Biosystems Science and Engineering, Indian Institute of Science, Bengaluru, India

**Keywords:** single-nucleotide polymorphism, two-component system, histidine kinase, *Mycobacterium tuberculosis*, evolutionary fitness, bacterial signal transduction, PhoR

## Abstract

The genome sequencing of *Mycobacterium tuberculosis*, the causative organism of tuberculosis, has significantly improved our understanding of the mechanisms that drive the establishment of infection and disease progression. Several clinical strains of *M. tuberculosis* exhibit single-nucleotide polymorphisms (SNPs), the implications of which are only beginning to be understood. Here, we examined the impact of a specific polymorphism in PhoR, the sensor kinase of the PhoPR two-component system. Biochemical analysis revealed reduced autophosphatase/ATPase activity, which led to enhanced downstream gene expression. We complemented *M. tuberculosis* H37Ra with the wild-type and mutant *phoPR* genes and characterized the strains in a cell line infection model. We provide an explanation for the low prevalence of the SNP in clinical strains (∼1%), as the mutation causes a survival disadvantage in the host cells. The study provides a rare example of selection of a signaling node under competing evolutionary forces, wherein a biochemically superior mutation aids bacterial adaptation within-host but has low fitness for infection and hence is not selected. Our study highlights the importance of accounting for such SNPs to test therapeutic and co-therapeutic methods to combat TB.

## Introduction

Sequencing of *Mycobacterium tuberculosis* H37Rv genome, a virulent laboratory strain, has revolutionized the research done on this pathogen world over (Cole et al., 1998). It revealed the presence of sizeable unknown gene sets, like serine–threonine protein kinases (STPKs) and other relatively small groups of typical bacterial signaling systems, the two-component systems (TCSs). Over the years, a large number of virulent clinical strains, non-human pathogenic strains, and non-pathogenic mycobacterial strains have been sequenced, aimed at unraveling what makes *M. tuberculosis* such a successful pathogen (Cole et al., 1998; Garnier et al., 2003; Zheng et al., 2008; Ioerger et al., 2010; Tekwu et al., 2014; Mohan et al., 2015; Manson et al., 2017; Borrell et al., 2019). The findings have revealed many differences; however, their contribution toward pathogenicity is not clear (Ioerger et al., 2010).

Given the pathogenic nature of *M. tuberculosis*, it is expected to face adverse environments within its host (Chai et al., 2018). To adapt and survive in changing environmental conditions, it uses signaling systems called TCSs, which regulate the expression of many genes in response to various environmental stimuli (Pang et al., 2007). TCSs are typically composed of a sensor kinase (SK) as the first component that senses the external stimulus and undergoes activation by an autophosphorylation event at a specific histidine (His) residue. The second component of a TCS is a response regulator (RR), which the SK activates by a unique phosphotransfer event on a conserved aspartate (Asp) residue (Stock et al., 2000; Bourret and Silversmith, 2010). The RRs are DNA-binding transcription factors that activate or repress the transcription of downstream genes and bring about adaptive changes in the bacterium. Among the 12 pairs of TCSs in *M. tuberculosis*, the PhoPR TCS is extensively studied and implicated in regulating various processes, such as virulence and growth upon infection (Pérez et al., 2001; Walters et al., 2006; Ryndak et al., 2014), lipid biosynthesis (Walters et al., 2006), hypoxia (Vashist et al., 2018), pH sensing, and adaptation (Johnson et al., 2015). It is known to regulate the expression of about 2% of the *M. tuberculosis* genome (Cimino et al., 2012). It is essential for survival and multiplication within macrophages (Ludwiczak et al., 2002), and thus, this operon has been considered to be a master regulator in *M. tuberculosis* (Ryndak et al., 2008). We analyzed the sequences of several clinical strains and sought single-nucleotide polymorphisms (SNPs) primarily in TCS genes to understand the evolutionary pressures subjected to TCSs.

Comparative sequence analysis of strains from various lineages of *M. tuberculosis sensu stricto* (L1–L4 and L7) and *Mycobacterium africanum* (L5 and L6), which belong to the human-adapted *M. tuberculosis* complex, that diverged from strains that infect animals and now cause the disease in humans (Comas et al., 2014), and *Mycobacterium bovis*, revealed close to ∼2,000 SNPs. These polymorphisms could be responsible for the physiological differences between them. Genomic differences among clinical strains have been associated with variations in protein and metabolite levels, post-translational modifications (Liu et al., 2014), drug susceptibility (Rouse et al., 1995), transcriptome (Homolka et al., 2010; Rose et al., 2013), and cell wall structure (Coscolla and Gagneux, 2014), along with virulence and infectivity. It is also observed that strains that have evolved more recently are more infectious, grow faster, and have lesser latency than their parental strains (Borrell et al., 2019).

A recent study showed that variations because of the passaging of laboratory strains used as reference pathogenic strains had impeded our understanding of the mycobacterial pathology (Ioerger et al., 2010). This includes the adaptation of H37Rv to laboratory conditions while remaining virulent in mice (Ioerger et al., 2010). It is believed that there are hot spots for insertions/duplications, deletions, or substitutions across the *M. tuberculosis* genome. Variants have also been observed in highly conserved TCS regions, such as a SNP in the DNA-binding domain of the *phoP* RR that attenuates the H37Ra strain (Wang et al., 2007; Chesne-Seck et al., 2008; Lee et al., 2008) and an SNP in the SK *phoR*, which shows functional and phenotypic changes in the clinical strain CDC1551 when compared with H37Rv (Schreuder et al., 2015), and yet another SNP in the *phoR* region of the *M. bovis* strain, which leads to pleiotropic changes in the production and secretion of lipids and affects virulence (Gonzalo-Asensio et al., 2014).

In the present study, we examined the role of one such polymorphism in the PhoR SK and its effect on mycobacterial signaling, virulence, and pathogenicity. Our findings demonstrate that the mutation enhanced the catalytic activity of the SK in both *in vitro* and *in vivo* conditions. The presence of the SNP in three of the 242 clinical strains examined suggests a poor evolutionary selection of this variant. We hypothesize that the mutant TCS is primed early and activates higher gene expression upon sensing of stimuli, leading to impaired survival of the mutation carrying bacilli during infection. This could explain the poor selection of the SNP in the wild. Overall, this study presents an example of the evolutionary benefits of temporally tuned signaling activity and suggests that similar competing forces may be at play with other TCSs.

## Materials and Methods

Chemicals, media, biochemicals, and protein reagents were obtained from Merck (Kenilworth, NJ, United States); protein marker was from Abcam (Cambridge, United Kingdom). Restriction enzymes were from Thermo Fisher Scientific (Waltham, MA, United States). Cloning and qRT primers were synthesized by Bioserve (Hyderabad, India); radioactive γ^32^P ATP (>4,000 Ci/mmol) was from BRIT-Jonaki (Hyderabad, India); fetal bovine serum (FBS) from Thermo Fisher Scientific (United States); TRIzol from TaKaRa (Seoul, South Korea); 0.1-mm zirconia beads from BioSpec Products Inc., (Bartlesville, OK, United States); iScript cDNA synthesis kit from Bio-Rad Laboratories (Hercules, CA, United States); DyNAmo Color Flash SYBR Green qPCR Kit from Thermo Fisher Scientific (United States); THP-1 monocyte cell line and H460 epithelial cell line from ATCC (Manassas, VA, United States); and H37Ra strain of *M. tuberculosis* from lab collection.

### *In silico* Sequence Analysis

The sequences of 19 clinical isolates from India, reported as part of a previous study (Ramaiah et al., 2019), along with 223 sequences from BioProject PRJNA235851 (Manson et al., 2017), were analyzed and aligned using T-COFFEE and CLUSTALW (Madeira et al., 2019) using the *phoR* gene sequence from H37Rv strain as a template. A variation from ***g****cc* to ***a****cc* at the 1,198 nucleotide of *phoR* gene was identified and named PhoR’ (A400T). The global frequency of the SNP was tested in the GMTV database (Genome-Wide *M. tuberculosis* Variation database)^1^ (Chernyaeva et al., 2014).

### Phylogenetic Tree Construction

The sequence alignment of *PhoR* gene was used to generate a radial phylogenetic tree using the maximum-likelihood method based on the Tamura–Nei model by MEGA. Bootstrap analysis was performed with 500 replicates, with all sites being informative and without gaps (38).

### Recombinant Plasmid Construction and Generation of Bacterial Strains

Cloning and overexpression of proteins were carried out in *Escherichia coli* strains DH10β and BL21 Arctic Express^TM^ (Agilent Technologies, Santa Clara, CA, United States) grown in LB medium with 100 μg/ml of ampicillin or 50 μg/ml of gentamycin, respectively. Primers used for PCR and cloning are listed in Supplementary Table 1. Recombinant plasmids used for protein overexpression are reported previously (Agrawal et al., 2015). For generating mycobacterial expression plasmid containing the PhoPR operon with its native promoter, the nucleotide region of the *phoPR* operon, along with a 500-bp upstream region, was PCR-amplified from H37Rv genomic DNA using specific primers. The amplicon was cloned in pCV125 vector, a mycobacterial single copy, and integrative expression vector with kanamycin resistance marker, at *Nde*I and *Hin*dIII restriction sites. The recombinant constructs generated were verified by DNA sequencing.

### Expression and Purification of Recombinant Proteins

The recombinant proteins were expressed and purified, as reported previously (Saini et al., 2002). In brief, *E. coli* cells containing expression plasmids for the SK and RR proteins were grown at 37°C in 200 ml of Terrific broth (TB) to an OD_600_ > 1.0 followed by induction with IPTG (0.1–1.0 mM). The culture was further grown for 15–20 h at 10°C–13°C for protein expression. Cells were harvested by centrifugation, and soluble 6 × His-tagged proteins were purified using Ni^+2^-NTA, as described previously (34). The pellets were resuspended in native lysis buffer (50 mM of Tris–Cl, pH 8, 300 mM of NaCl, and 10% glycerol) with 1 mM of phenylmethylsulfonyl fluoride (PMSF) and 1 mM of benzamidine and sonicated on ice for 5–10 min at 25% amplitude (pulse on 3 s, pulse off 2 s). The lysate was centrifuged at 14,000 *g* for 30 min at 4°C. The supernatant containing the protein was passed through the Ni^+2^-NTA column for about 120 min with intermittent shaking on a pre-equilibrated with native lysis buffer at 4°C for 15 min. The unbound proteins were discarded as flow-through, and the column was washed with wash buffer A (25 mM of Tris–Cl, pH 8.0, 500 mM of NaCl, 25 mM of imidazole, and 10% glycerol) and wash buffer B (25 mM of Tris–Cl, pH 8.0, 500 mM of NaCl, 50 mM of imidazole, and 10% glycerol). The bound protein was eluted out with elution buffer (25 mM of Tris–Cl, pH 8.0, 500 mM of NaCl, 250 mM of imidazole, and 10% glycerol).

### Dialysis and Storage of Purified Proteins

The eluted fractions of the proteins with the highest yield determined by Bradford assay were pooled together and dialyzed against dialysis buffer I (50 mM of Tris–Cl, pH 8.0, 50 mM of NaCl, 1 mM of DTT, and 10% glycerol) for 6–12 h and then with dialysis buffer II or storage buffer (50 mM of Tris–Cl, pH 8.0, 50 mM of NaCl, 0.1 mM of DTT, and 50% glycerol) overnight. The concentration of the purified proteins after dialysis was determined by Bradford assay using bovine serum albumin (BSA) as standard. The purity of the proteins was checked on sodium dodecyl sulfate–polyacrylamide gel electrophoresis (SDS-PAGE) and stored at −20°C.

### Circular Dichroism Spectroscopy

PhoR wt and PhoR’ (A400T) proteins were subjected to circular dichroism spectroscopy using JASCO J-810 Spectropolarimeter. Spectra for SK protein (in 1 × PBS) were analyzed between wavelength 190 and 300 nm to record protein secondary structures. The K2D3 software was used to analyze the plots.

### Phosphorylation Assays

Autophosphorylation assays were performed as described previously (Agrawal et al., 2015). Briefly, 50 pmol of the purified SKs [PhoR wt and PhoR’ (A400T)] were autophosphorylated in kinase buffer (50 mM of Tris–Cl (pH 8.0), 50 mM of KCl, and 20 mM of MgCl_2_), 100 μM of ATP, and 2 μCi of γ^32^P-ATP (>4,000 Ci/mmol) for 60 min at 30°C. The reaction was terminated using a 1 × SDS-PAGE buffer and resolved on a 12.5% SDS-PAGE gel. The gels were washed and exposed to a phosphor screen (Fujifilm, Tokyo, Japan), followed by imaging with Typhoon phosphorimager (GE Healthcare, Chicago, IL, United States). Images were adjusted for brightness and contrast with the Microsoft image editing tool, and quantitative densitometric analysis of the autoradiograms was done using ImageJ software. The first time point’s signal was considered 100%, and relative levels of phosphorylation over time with the wild-type (WT) and mutant SKs were determined to quantify the effects of the mutation. For statistical analysis, significance (*p*-values) were calculated regarding SK∼P levels in the autophosphorylation reaction. In the case of the autophosphorylation of the SK in the presence of the PhoR inhibitor, ethoxzolamide (ETZ) (Johnson et al., 2015), the WT and mutant SKs were incubated for 2 h with increasing concentrations of the inhibitor in kinase buffer (described above) at 30°C. The reaction was terminated using a 1 × SDS-PAGE loading buffer, loaded on an SDS-PAGE gel, and processed as described above. In the phosphotransfer assay, 150 pmol of the RR diluted in kinase buffer was added to the autophosphorylation reaction, containing 50 pmol of phosphorylated SK (for 60 min) and incubated for 60 min, and the reaction was terminated using a 1 × SDS-PAGE loading buffer. The samples were loaded on an SDS-PAGE gel and processed as described above.

### ATP Hydrolysis and ^32^IP Release Assay by Thin-Layer Chromatography

In the phosphotransfer reaction, 150 pmol of the RR diluted in kinase buffer was added to the autophosphorylation reaction of 10 μl volume, containing 50 pmol of phosphorylated SK, for the indicated times. The reaction was terminated using 50 mM of EDTA. The amounts of labeled ^32^P released and residual ATP in the reaction were determined by thin-layer chromatography (TLC) on polyethyleneimine-cellulose plates using 2 M of HCOOH and 2 M of LiCl (2:1) as the mobile phase; the dried plates were exposed to a phosphor screen and scanned with Typhoon phosphorimager. A quantitative comparison for the amount of ^32^iP released was made by normalizing the later time points in the presence of the RR to the ^32^iP generated by the SK alone.

### Electrophoretic Mobility Shift Assay

A 500-bp region corresponding to the *aprA* promoter region was PCR-amplified from *M. tuberculosis* H37Rv genomic DNA template using specific primers (Supplementary Table 1). The PCR products were purified and end-labeled with γ^32^P-phosphate using T4 polynucleotide kinase (Thermo Fisher Scientific, United States) as per the manufacturer’s protocol. The labeled fragments were purified and used for electrophoretic mobility shift assay (EMSA), by incubating with the indicated amount of PhoP protein for 45 min at 25°C in the binding buffer (25 mM of Tris–Cl, pH 8.0, 20 mM of KCl, 6 mM of MgCl_2_, 0.10 mg/ml of BSA, 0.5% glycerol, 1 mM of DTT, 0.5 mM of EDTA, and 1 μg of poly dI.dC). SK proteins were autophosphorylated with 5 mM of ATP and incubated with PhoP to obtain PhoP∼P. The reaction mixtures were resolved on a 4% native polyacrylamide gel (29.5:0.5) and pre-equilibrated for 1–2 h at 80 V in 0.5 × Tris–borate EDTA buffer at 4°C. Electrophoresis was performed at 4°C at 80 V for 2–3 h. The DNA–protein complexes were visualized by phosphorimaging, as described above.

### Analysis of *aprA* Promoter-Reporter Activity

Cultures of *M. tuberculosis H37Ra* containing either the pCV125 empty vector or the WT or mutant *phoPR* construct were electroporated with the *aprA*’:GFP, *smyc*’:mCherry (obtained as a kind gift from Prof. R. Abramovitch, United States) promoter-reporter construct (Abramovitch et al., 2011). The strains were grown in 10 ml of 7H9 media buffered to pH 5.5 for induction and kept at pH 7.4 for uninduced conditions with an initial OD_600_ of 0.05 for 12 days for PhoR activation experiments. At each time point, samples from each culture were taken in a 96-well flat black clear-bottom plate (Corning Inc., New York, NY, United States). Green fluorescent protein (GFP) and mCherry fluorescence ratios were measured using the Tecan Infinite M1000 plate reader (Tecan, Grödig, Austria) in duplicates along with OD_600_. On the 12th day, cultures were fixed in 4% paraformaldehyde and mounted on glass slides in 10% glycerol. The relative fluorescence for ∼300 bacilli from each sample was quantified by microscopy analysis from three individual experiments. In infection experiments (below), for *in cellulo* analysis, H460 lung epithelial cell line (ATCC) was infected at a multiplicity of infection (MOI) of 1:10; infected cells were fixed and imaged at 4 h post-infection using the Olympus IX83 fluorescence microscope to measure the relative GFP by mCherry ratios from the infected bacilli.

### RNA Extraction and Quantitative Gene Expression Analysis

Cultures of *M. tuberculosis H37Ra* containing either the pCV125 empty vector backbone alone or either the WT or mutant *phoPR* construct were grown exponentially to an OD_600_ of 0.8 and harvested. For acid induction experiments, cultures were grown in 10 ml of 7H9 buffered to pH 5.5 (using HCl) for induced cultures and pH 7.4 for uninduced cultures. The pellets were resuspended in QIAzol Lysis reagent (Qiagen, Hilden, Germany), mixed with zirconia silica beads, and disrupted using a mini bead beater, following which RNA was precipitated using ethanol. The RNA obtained with OD_260_/OD_280_ ratios ≥ 2 was treated with DNaseI, and 500 ng of the total RNA was reverse transcribed using random hexamers and iScript reverse transcriptase (Bio-Rad, United States) as per the manufacturer’s protocol. Gene-specific qRT-PCR was performed using DyNAmo Color Flash SYBR Green qPCR Kit (Thermo Fisher Scientific, United States) in Roto-GeneQ cycler (Qiagen, Germany), using 0.5 μl of the cDNA synthesized per 10-μl reaction with primers from previously published studies as indicated in Supplementary Table 1. The calculated threshold cycle (Ct) value for each gene was normalized to 16S rRNA followed by that of the gene of interest in the strain containing vector-only to determine fold change. The expression analyses were performed using three independent biological replicates.

### Macrophage Cell Line Infections

Infection experiments were performed as previously described (Estrella et al., 2011). Briefly, THP-1 monocytes (ATCC) differentiated into macrophages with 20 nM of phorbol 12-myristate-13-acetate were infected at an MOI of 1:10 with the three strains of H37Ra (pCV125 empty vector backbone alone or the WT or mutant *phoPR* construct). The bacteria were allowed to infect cells for 4 h (taken as the time point of invasion), after which cells were washed with PBS thrice and either lysed with Triton X-100 and plated after serial dilutions on Middlebrook 7H11-OADC plates for colony enumeration or replenished with fresh antibiotic-free Roswell Park Memorial Institute (RPMI) medium to be lysed and plated at a later time point as indicated. In the experiments with PhoR inhibitor, cells were pretreated with 80 μg ETZ, which was replenished every 2 days (wherever needed), and equal volumes of DMSO was added to untreated cells, followed by lysing of the cells and plating as described above. This was repeated to obtain three biological replicates (Johnson et al., 2015).

### Measurement of Nitrite Production

The nitrite produced by infected THP-1 cells was measured as described previously using the Griess reagent (37). Briefly, the supernatants of uninfected and infected cells were collected at indicated time points and then incubated with the Griess reagent in a 1:1 proportion at room temperature for 10 min, and the absorbance was measured at 540 nm in the Tecan Infinite M1000 plate reader. The nitrite concentration was determined using a standard curve plotted with different sodium nitrite concentrations and represented as μM/10^5^ cells per well, and normalized to the uninfected 4-h time point of invasion and blanked with the cell-free medium. This was performed to get three biological replicates, and *p*-values (statistical significance) were calculated the Mann–Whitney test.

### Statistical Analyses

Statistical analyses for significance were performed using Student’s *t*-test, one-way ANOVA test, and two-way ANOVA test (for time-course experiments) between control and experimental sets and induced and uninduced sets (wherever applicable). For all experiments, the number of independent biological replicates used is indicated by “*n*.”

## Results

Clinical isolates of *M. tuberculosis* harbor several SNPs leading to variations in their sequences compared with laboratory strains. Although the changes brought about by sequence variations in various bacterial genome regions have been characterized previously, TCS regions have seldom been studied. To check the effect of such polymorphisms on the signaling and the interactions between the bacterium and its host, we analyzed the gene sequence of the *phoPR* TCS.

### An Single-Nucleotide Polymorphism in the HATPase Domain of the Sensor Kinase PhoR Is Found in Some Clinical Strains of *Mycobacterium tuberculosis*

**FIGURE 1:**
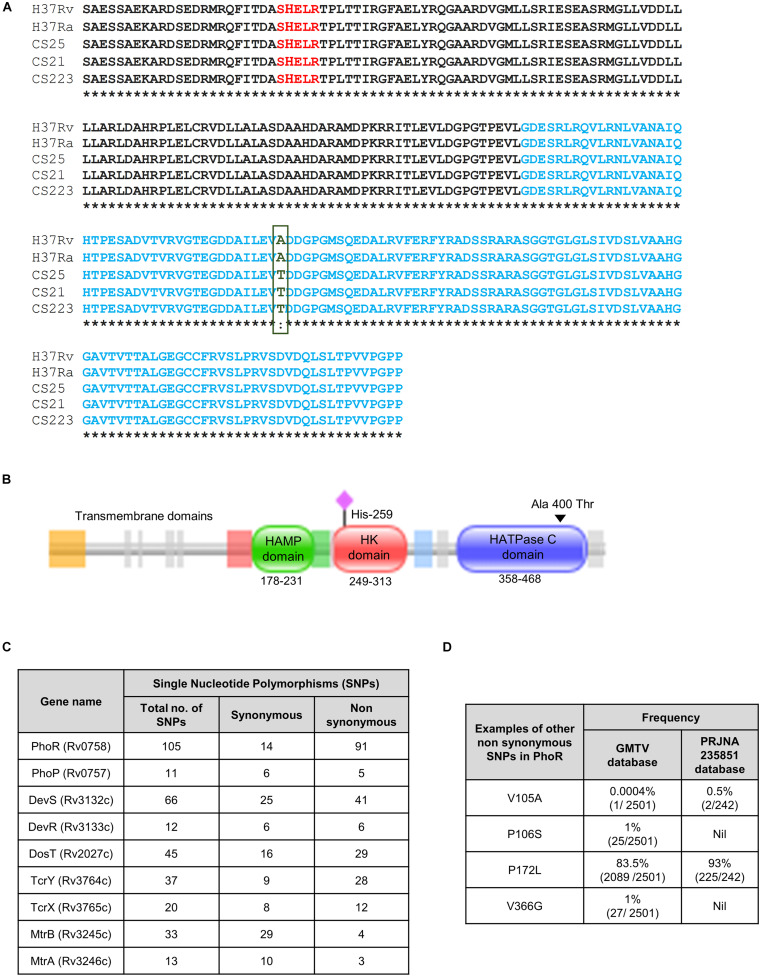
Single-nucleotide polymorphisms (SNPs) in the sensor kinase PhoR and other two-component systems (TCSs) in the clinical strains of *Mycobacterium tuberculosis*. **(A)** Sequence alignment of the sensor kinase (SK) protein PhoR, with H37Rv as the reference strain aligned with H37Ra and three clinical strains. HATPase domain is marked in blue with the mutation from alanine to threonine at 400 marked in green. CS25, CS21, and CS223 are the numbers assigned to three of the 223 clinical strains that carry this mutation analyzed in this study. **(B)** Pfam analysis of the SK protein PhoR from H37Rv, depicting the presence of three distinct domains, the HAMP domain (amino acids 178–231), the histidine kinase (HK) domain (amino acids 249–313), and the HATPase domain (amino acids 358–468). The predicted transmembrane domains are depicted to the left of these domains. The black triangle marks the mutation in the HATPase domain, and the pink diamond marks His^259^, the phosphorylation site. **(C)** Table showing the frequencies of synonymous and non-synonymous SNPs in various TCS proteins wrt H37Rv as reference genome reported in the GMTV database. **(D)** Table showing the frequencies of SNPs that are found in the PhoR sensor kinase in GMTV database and PRJNA235851 database.

We analyzed the sequence of *phoR* in 242 mycobacterial strains from BioProject PRJNA235851 in National Center for Biotechnology Information (NCBI) (Ramaiah et al., 2019) with a combination of drug-sensitive/resistant, polydrug- and multidrug-resistant cohort of a South Indian population (Ramaiah et al., 2019) with H37Rv as the reference sequence to identify various SNPs, and we found one that translated into a non-synonymous, missense mutation in the protein. This SNP changed alanine to threonine residue (A400T) at the 400th position, in the HATPase domain (Figures 1A,B). The SNP was found in only three strains of the genome sequences analyzed from the cohort (Figure 1A). A schematic phylogenetic tree drawn with the PhoR sequence alignment, for the genomes analyzed, shows that the strains with the mutation A400T group separated from the rest of the strains (Supplementary Figure 1). We also analyzed the genome sequence repository database GMTV, which consists of 2,501 clinical genome sequences that have been aligned for mutations in different regions of the bacteria; however, this particular SNP was not reported there. Subsequent analyses of the frequencies of other SNPs in the SK PhoR (Figure 1C) revealed no particular hot spots for any SNPs in the gene.

Interestingly, other TCSs in these strains have SNPs in the various regions with varied frequencies (Figure 1D). The low frequency of the mutation in our study and absence in the GMTV database made us explore the changes brought about by this mutation A400T on the catalytic activities of the SK PhoR.

### A400T Substitution in the Kinase Domain Alters PhoR Phosphatase Activity *in vitro*

**FIGURE 2:**
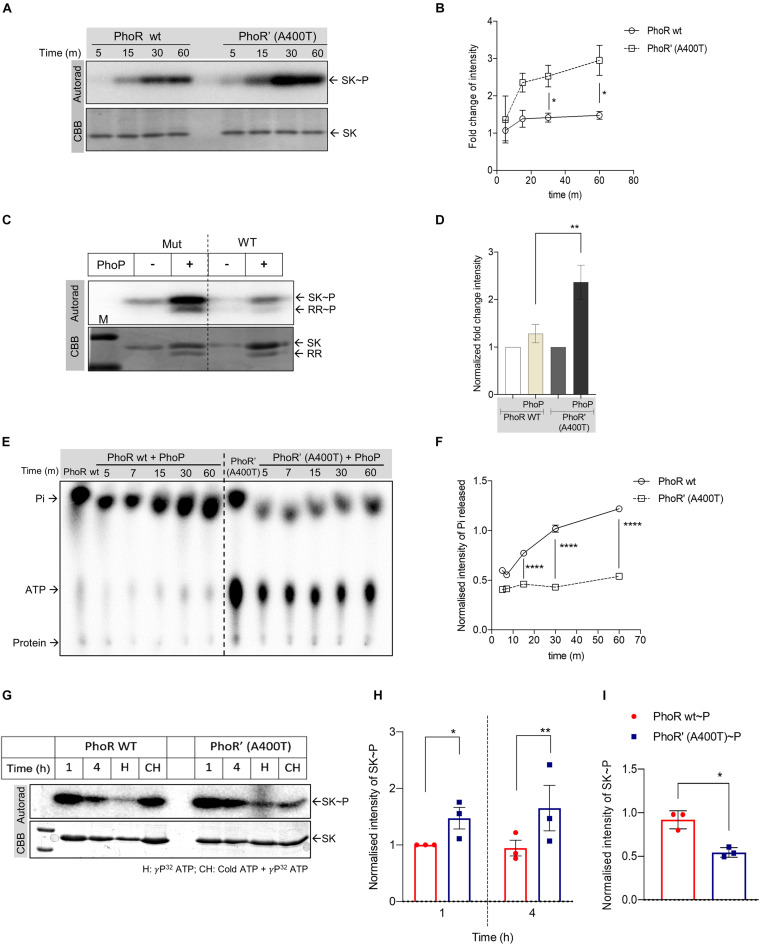
Analysis of the impact of A400T single-nucleotide polymorphism (SNP) on the biochemical activities of the sensor kinase PhoR. **(A)** Autophosphorylation time-course analysis. The assay was performed using wild-type (left) and mutated PhoR’ A400T (right) for indicated time points. Top panel, autoradiogram; bottom, Coomassie Brilliant Blue (CBB)-stained gel. **(B)** Quantitative measurement of autophosphorylated wild-type or mutated PhoR proteins at various time points as shown in panel **(A)**. The signal recorded for both wild-type and mutant proteins at the first time point was taken as 1, and the rest of the time points were normalized to it (*n* = 3). **(C)** Phosphotransfer analysis to analyze the effect of the mutation in the sensor kinase (SK) PhoR. The assay was performed using mutated (left) or wild-type PhoR (right) with wild-type response regulator (RR) protein PhoP. Top panel, autoradiogram; bottom panel, CBB-stained gel. **(D)** Quantitative analysis of signals of phosphorylated wild-type PhoR and mutated PhoR’ A400T protein at 1-h post-phosphotransfer. The signal recorded for wild-type and mutant proteins without RR was taken as 1, and the signal in the presence of RR at various time points was normalized to it. **(E)** Thin-layer chromatography (TLC) analysis of phosphotransfer time-course reaction to determine the effect of the mutation on the phosphatase activity. The assay was performed using wild-type (left) or mutated PhoR’ A400T (right) protein in the presence of the RR PhoP as described in “Materials and Methods” section. **(F)** Quantitative measurement of the amount of inorganic phosphate (Pi) generated by PhoR wt (represented by solid line, circles) or mutant PhoR’ (represented by dotted line, squares) in the presence of PhoP proteins at various time points as shown in panel **(E)**. The Pi generated by the SK alone post 2 h of autophosphorylation was taken as 1, and the subsequent time points are normalized to it (*n* = 3). **(G)** Kinase and phosphatase activity analysis of the PhoR proteins. The autophosphorylation assay was performed for indicated time points for wild-type (left) and mutant (right) PhoR protein, with conditions as indicated. Top panel, autoradiogram; bottom panel, Coomassie Brilliant Blue (CBB). Lanes marked 1 and 4, autophosphorylation reaction for 1 and 4 h, respectively; lane H, samples analyzed after heating at 95°C for 5 min; and lane HC, proteins first incubated with cold ATP for 2 h followed by ^32^P[ATP] for two additional hours before analysis (hot-chase analysis). **(H)** Quantitative analysis of autophosphorylation of wild-type and mutant PhoR proteins at 1- and 4-h incubation, as shown in panel **(G)**. The signal from wild-type phosphorylated PhoR at 1 h is taken as 1, and the rest of the points are normalized to it (*n* = 3). The signal intensity from the autorad was normalized to protein amount (as per CBB staining). **(I)** Quantitative analysis of SK phosphorylation post-incubation with cold ATP for 2 h followed by phosphorylation with ^32^P labeled ATP for two additional hours (hot-chase analysis), as shown in panel **(G)**, with lanes labeled CH. The signals recorded for wild-type and mutant PhoR proteins were normalized to the amount of protein loaded (*n* = 3). The *p*-value was calculated based on the amount of SK∼P formed [for panels **(C,E)**] and the amount of Pi generated in the presence of the RR (PhoP) with respect to SK∼P [for panels **(E,F)**]. n represents the number of biological replicates used in the experiments. *p*-values, * ≤ 0.05, ** ≤ 0.01, *** ≤ 0.001, and **** ≤ 0.0001, were determined by two-way ANOVA for the autophosphorylation and time-course experiments and Student’s *t*-tests for the phosphotransfer experiment.

We introduced the mutation in the cytosolic C-terminal domain of the SK protein PhoR (Agrawal et al., 2015) by site-directed mutagenesis and purified both WT and mutant proteins. Predicted structure analysis of the HATPase and kinase domain of the WT and mutant protein revealed no major structural differences (Supplementary Figure 2A), and circular dichroism analysis also revealed no structural perturbations between the purified proteins (Supplementary Figure 2B), allowing us to proceed with biochemical analysis of the mutant protein. We performed autophosphorylation analysis for the WT and mutant SK proteins. The proteins were incubated with γ^32^P-labeled ATP, and their phosphorylation status was examined. We observed enhanced autophosphorylation for the mutant compared with the WT PhoR protein at all time points (Figures 2A,B). Next, we examined the ability of the phosphorylated WT and mutant SKs to transfer the phosphoryl moiety to the RR protein PhoP. A stable and higher phosphotransfer was observed with the mutant PhoR’ (A400T) protein (Figure 2C), and on quantitation, the amount of RR∼P was also detected to be higher (Figure 2D).

The faint signal on the WT PhoR (Figure 2C, right) suggested an inherent phosphatase activity in the PhoR SK, which seems to be reduced in mutant SK as well as in the presence of RR PhoP. While the presence of a phosphatase activity in SK toward RR∼P is widely reported and is attributed to the DHp domain (Xing et al., 2017), the presence of an autophosphatase activity of SK is rarely reported (Dubey et al., 2016). Such an activity would result in dephosphorylation of the SK∼P, releasing free ^32^iP. We measured the concentration of ^32^iP species released through TLC analysis by incubating phosphorylated WT or the mutant SK in the presence or absence of the RR PhoP. The intensities were normalized to those with SK alone, and the relative ^32^iP and ATP signal intensities of the spots at various time points were noted in the presence of the RR PhoP. Figure 2E (lanes 1 and 7) indicates two things: first, both proteins have an ATPase or autophosphatase activity, as evidenced by the amount of ^32^iP released; and second, the mutant kinase exhibited a reduced auto-dephosphorylation ability compared with the WT protein.

We concomitantly recorded higher levels of residual ATP for the mutant PhoR’ (A400T) protein (Figure 2E, right panel; lanes 7–12), compared with the WT kinase (Figure 2E, left panel; lanes 1–6). Figure 2F shows a significant reduction in the amount and rate of ^32^iP released for the mutant protein. Taken together, the data indicate that the mutant SK PhoR’ (A400T) protein is catalytically more active than the WT PhoR by virtue of stable phosphotransfer and a decreased phosphatase activity.

To further test this, we analyzed the autophosphorylation of the WT and mutant PhoR under different conditions. First, we examined the phosphorylation over an extended period of 4 h, and this revealed that, while 1 h of phosphorylation is sufficient to generate a peak signal for the proteins, significant phosphorylation was also detected at 4 h; and as anticipated, it was higher in the mutant protein (Figure 2G, lanes 1 and 2 vs. lane 5 and 6; and Figure 2H). Interestingly, heating of the reaction mix before analysis led to a significant phosphorylation reduction (Figure 2G, lanes 3 and 8). This could be due to the loss of SK–ATP complex [an intermediate formed during the autophosphorylation reaction, which yields SK∼P (Sankhe et al., 2018)], allowing signal only from SK∼P. As expected, the mutant showed a higher signal than the WT, indicating that the mutant retains a higher phosphoryl signal.

In a parallel reaction aimed to examine phosphoryl group turnover, the SKs were first incubated with cold ATP for 2 h, followed by the addition of ^32^P labeled ATP for two additional hours (hot-chase analysis). This experiment’s premise was that the protein that possesses higher ATP turnover would be labeled more with the ^32^P in step 2. In agreement with the observations above, we recorded higher labeling for WT PhoR protein, confirming that the mutant PhoR has a reduced autophosphatase activity (Figure 2G, lanes 4 and 9; and Figure 2I).

### PhoR’ A400T Enhances the DNA-Binding Ability of RR PhoP on Target Gene *aprA*

The *phoPR* TCS upon activation regulates close to 2% of *M. tuberculosis*’s total genome, including a diverse set of genes involved in lipid synthesis, secretion of virulence factors, and genes regulating acid stress survival (14). *apr* genes form a part of the regulon of this TCS, and their expression increases at low pH when the PhoPR TCS is activated (Abramovitch et al., 2011).

**FIGURE 3:**
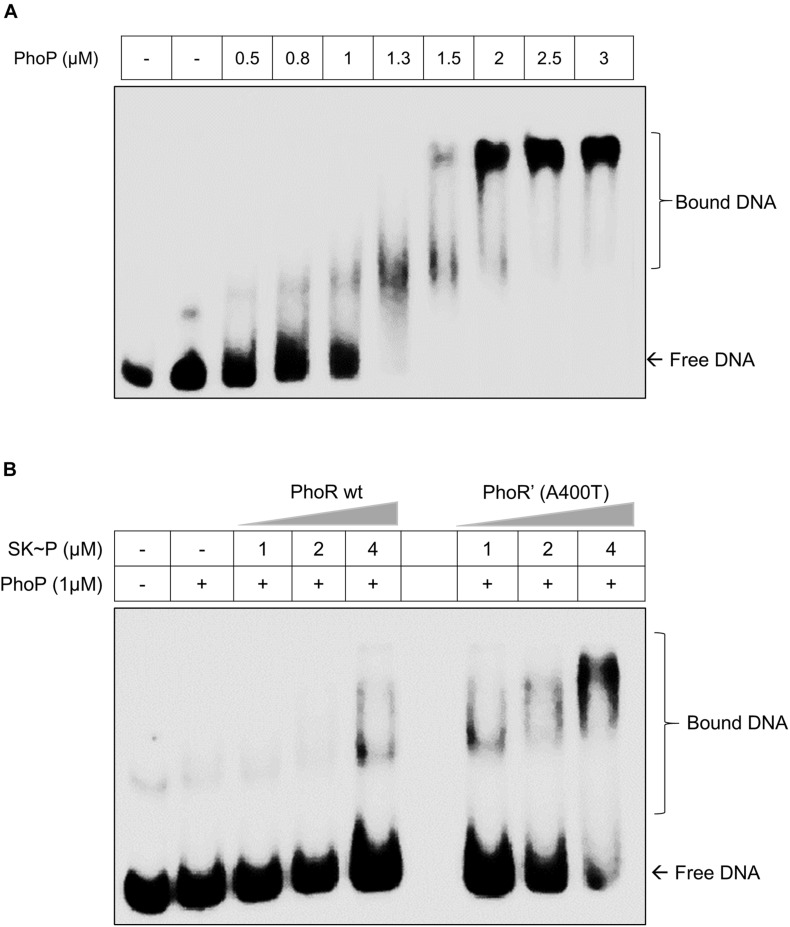
Effect of the PhoR single-nucleotide polymorphism (SNP) on the DNA-binding activity of response regulator (RR) PhoP. **(A)** Titration of RR PhoP protein to determine a concentration that shows low binding of unphosphorylated PhoP to the promoter region of the *aprA* gene. The DNA fragment corresponding to the *aprA* promoter was incubated with an increasing concentration of PhoP protein (as indicated) and tested by electrophoretic mobility shift assay (EMSA). The RR RegX3 was used as a negative control for non-specific binding (lane 2 marked –ve). **(B)** A comparative analysis of PhoP∼P (1 μM) binding to *aprA* promoter DNA as a function of phosphorylation mediated by the wild-type [PhoR wt, left] or mutant [PhoR’ (A400T), right] sensor kinase (SK) proteins at different concentrations. For all experiments, *n* = 3, where n represents the number of biological replicates.

The decreased phosphatase activity of the PhoR’ (A400T) would effectively increase the availability of the phosphorylated kinase and consequently the activated RR PhoP. Thus, we examined the DNA-binding ability of phosphorylated PhoP generated through either PhoR wt or PhoR’ (A400T) mutant proteins to the promoter region of *aprA* gene by EMSA. A 500-bp region upstream of *aprA* gene labeled with γ^32^P-labeled ATP was used as a probe for these experiments. Although the RR alone at higher concentrations binds to the promoter, we used a lower optimized concentration (1 μM), which did not show binding to the promoter in our assays (Figure 3A). When the probe was incubated with a mixture of SK and RR proteins in the presence of ATP, an increased mobility shift with increasing amounts of SK was recorded (Figure 3B). We also confirmed that the unphosphorylated SK, either WT or mutant, does not bind to the *aprA* promoter by themselves (Supplementary Figure 3). These findings suggest that an increase in autophosphorylation and phosphotransfer ability of the PhoR’ (A400T) protein would induce higher downstream gene expression. This improved activation of the *phoPR* TCS regulon led us to examine changes brought about *in vivo* by this polymorphism.

### *In vivo* Analysis of Downstream Gene Expression in Wild-Type or Mutant PhoR Complemented Strains of *Mycobacterium tuberculosis* H37Ra

**FIGURE 4:**
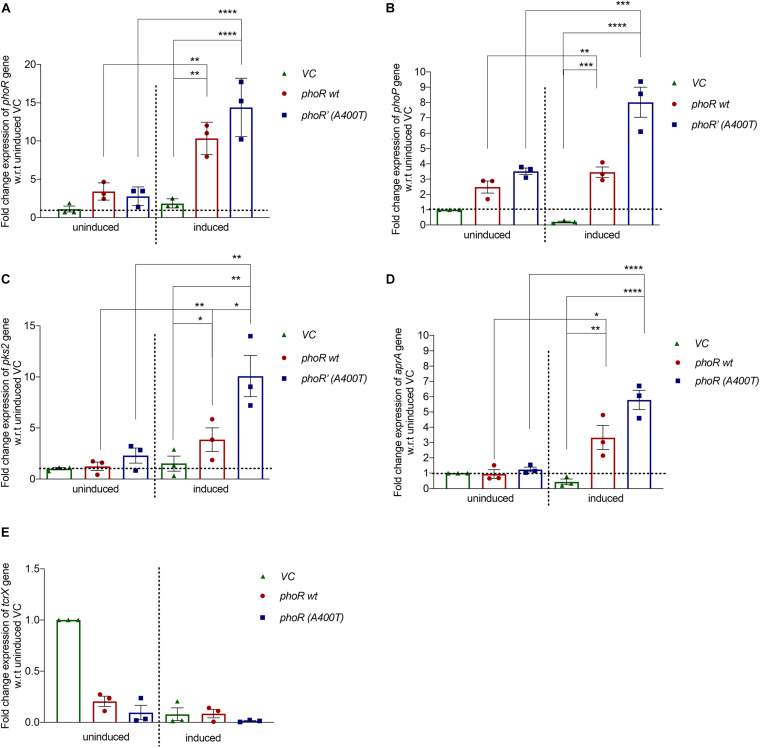
Expression analysis of downstream genes in *Mycobacterium tuberculosis H37Ra* strain complemented with wild-type or mutant *phoPR* operon. Relative mRNA expression analysis from H37Ra strains carrying a single integrated copy of either vector alone (pCV125) or with the wild-type or mutant *phoPR* operon, grown in Middlebrook 7H9 medium at pH 7.2 (uninduced) or at pH 5.5 (acid-induced). The expression was normalized to the levels of 16S rRNA, followed by the levels of the specific gene in the uninduced condition in strains carrying the empty vector (vector control). For all panels: left, uninduced (pH 7.2); and right, induced (pH 5.5). Expression analysis for **(A)** *phoR* gene, **(B)** *phoP* gene, **(C)** *pks2* gene, **(D)** *aprA* gene, and **(E)** *tcrX* is shown. The primer sequences for all the genes examined were obtained from previously published studies and listed in Supplementary Table 1. For all experiments, *n* = 3, where n represents the number of biological replicates. *p*-values, * ≤ 0.05, ** ≤ 0.01, *** ≤ 0.001, and **** ≤ 0.0001, were determined by one-way ANOVA.

To check the mutant PhoR protein’s effect *in vivo*, we cloned the *phoPR* operon from *M. tuberculosis* H37Rv with its native promoter (∼500 bp upstream) in an integrative single-copy mycobacterial shuttle vector, pCV125. We introduced the mutation (A400T) in the WT construct and electroporated both the WT and mutant plasmids in H37Ra. We used H37Ra since it has a functionally defective PhoPR system and partially regains its virulence and persistence upon complementation with PhoP from H37Rv (Lee et al., 2008), making it a suitable model system for our studies. We checked the expression levels of *phoR* and *phoP* genes in the complemented strains by RT-PCR. Similar levels of *phoR* transcripts were observed in both strains under basal conditions at pH 7.2. At low pH (5.5), known to induce expression of *phoPR* TCS, we observed enhanced *phoR* expression in WT strain (∼10-fold) as well as mutant strain (∼12-fold) (Figure 4A). We also examined for change in the *phoP* transcript levels. As shown in Figure 4B, *phoP* expression significantly increased in the mutant strain under inducing conditions (∼8-fold) as compared with a ∼1.5-fold increase observed in the WT strain. Since no significant changes were observed with the mutant strain under uninduced conditions, we rationalized that the upregulation of *phoP* gene resulted from the increased phospho-signaling and downstream autoregulatory effect of the RR PhoP.

We also analyzed the expression levels of a specific downstream target gene, *pks2* (polyketide synthase for SL-1 biosynthesis) (Walters et al., 2006). Expectedly, we observed a ∼10-fold increase in *pks2* transcripts in the mutant strain and a fourfold increase in the WT strain under inducing conditions (Figure 4C, left). These observations collectively validate the effect of an improved activation of the PhoP RR through phosphorylation by the PhoR’ mutant SK on upregulation of PhoPR regulon (Figures 4A–C, right). We also checked the expression of *aprA* gene, another downstream gene of the PhoPR TCS, and found that it was induced under conditions of low pH, as expected with the strains expressing the WT PhoR showing a ∼3-fold induction and the mutant PhoR’ strain sixfold induction relative to the vector control. More importantly, the induction was significantly higher (twofold) in mutant PhoR’ carrying bacilli compared with the WT PhoR strains (Figure 4D, right); as a control, we analyzed the expression of *Rv3764* (*tcrX*) gene and found no significant change in its expression levels among the strains in any condition (Figure 4E).

### Expression of *aprA* Is Induced to a Greater Extent in PhoR’ Mutant Strain at Acidic pH

**FIGURE 5:**
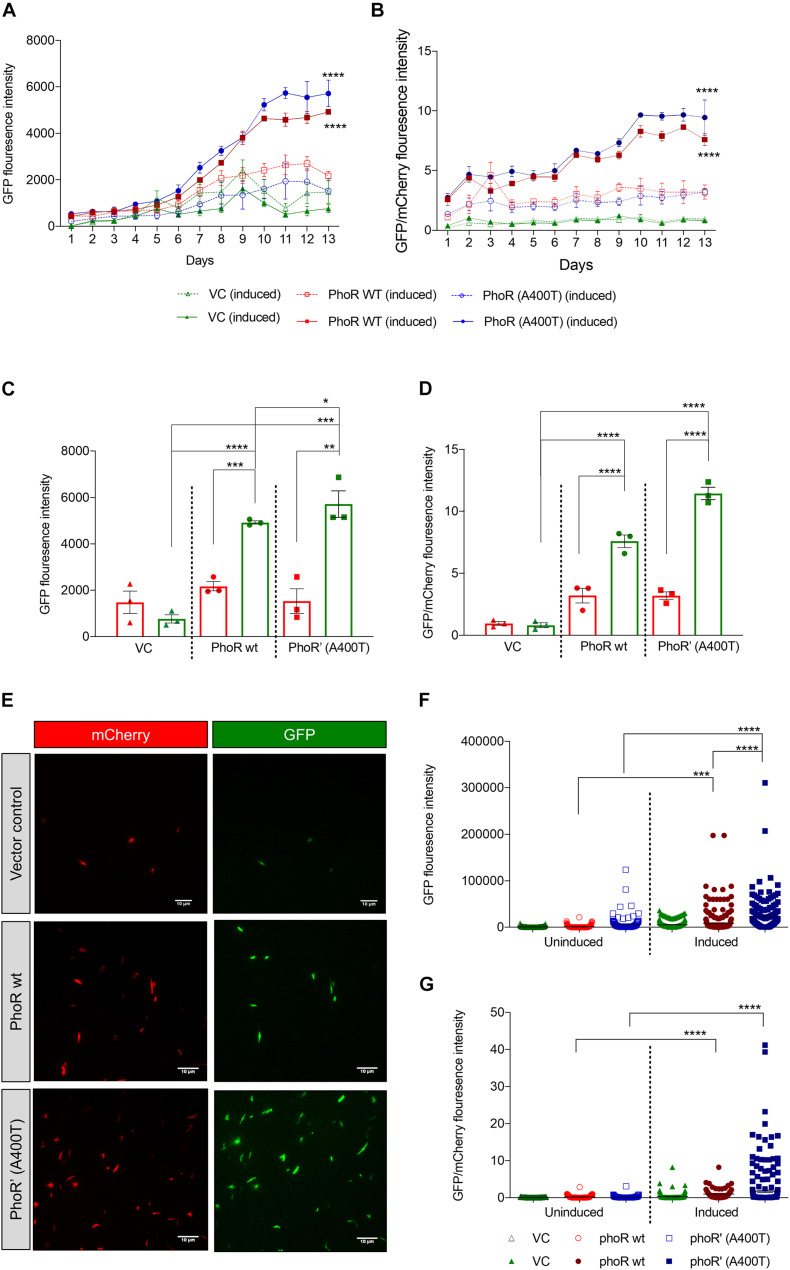
Expression analysis of PhoPR regulated *aprA* gene using promoter-reporter system in *Mycobacterium tuberculosis* H37Ra. **(A)** Green fluorescent protein (GFP) fluorescence in H37Ra strains carrying *aprA* promoter-reporter plasmid and integrative vector pCV125 alone or containing wild-type or mutant *phoPR* operon in cultures grown at pH 7.2 or at pH 5.5. The solid lines show GFP fluorescence at pH 5.5, and the dotted lines that at pH 7.2 over 13 days. **(B)** Analysis of the relative fluorescence ratio of GFP/mCherry, at pH 5.5, solid lines; and at pH 7.2, dotted lines (triangles, vector control; circles, *phoR* wt; and squares, *phoR*’ (A400T) mutant). **(C)** Quantitation of GFP fluorescence in cultures (as indicated) at day 12 at pH 5.5 (green, induced) and at pH 7.2 (red, uninduced). **(D)** Quantitation of the relative fluorescence ratio of GFP/mCherry from induced and uninduced cultures at day 12 at pH 5.5 (green) and at pH 7.2 (red). **(E)** Fluorescence microscopy images showing GFP expression in cultures at day 12 in indicated strains. Left panel, mCherry; right panel, GFP. **(F)** Quantitation of GFP fluorescence and **(G)** relative fluorescence by GFP/mCherry ratios by fluorescence microscopy in various strains and conditions (as indicated) from ∼300 individual bacilli. *n* = 3, where n represents the number of biological replicates used in the experiments. *p*-values * ≤ 0.05, ** ≤ 0.01, *** ≤ 0.001, and **** ≤ 0.0001, were determined by two-way ANOVA.

Given that we found differences in the induction and expression of Pho regulon in the WT and mutant strains in response to pH stimulation, we introduced a promoter-reporter plasmid containing *aprA* promoter-driven eGFP2 and constitutively expressed mCherry (Abramovitch et al., 2011). The objective was to measure GFP fluorescence as a readout of the activation of the TCS. The strains carrying WT or mutant PhoPR showed induction of an *aprA* promoter activity over 12 days at acidic pH of 5.5, unlike at pH 7.2 (Figure 5A). A 12-day time point was chosen, as it has been previously reported to be reliable time point to record acid-mediated induction (Abramovitch et al., 2011). As anticipated, the strain-carrying mutant PhoPR TCS demonstrated a higher expression of *aprA GFP* over time, with a significant difference at day 12 compared with the strains with WT PhoPR and the vector control (Figure 5C). When normalized to mCherry, we observed a significantly higher expression of *aprA* in the mutant than the WT (Figures 5B,D). A similar observation of expression differences in these strains at day 12 post-acid induction was made by microscopic analysis (Figure 5E). Quantification of fluorescence from ∼300 bacterial cells expressing both GFP and mCherry per strain (Figures 5F,G) confirmed that the mutant strain has a higher *aprA* promoter activity.

To verify that the activation of *aprA* at low pH is through the *phoPR* TCS, we used an inhibitor of this TCS ETZ, previously reported to inhibit the *phoPR* system (Johnson et al., 2015). After 24 h of treatment with 80 μM of ETZ (Supplementary Figure 4A) at day 12 post-induction, reduction in the expression of *aprA* fluorescence was recorded (Supplementary Figures 5A,B) in the strain carrying the WT copy. However, the reduction was lesser in the strain complemented with mutant PhoR, showing that the SNP in clinical strains could potentially alter their response to drugs. To determine where this inhibitor could be acting, we checked the autophosphorylation of PhoR wt and PhoR’ (A400T) protein in the presence of different concentrations of the inhibitor. We observed inhibition of phosphorylation for both WT and mutant SK proteins. This inhibition was lesser in the mutant than the WT (Supplementary Figures 4C,D), suggesting that the inhibition of the TCS PhoPR observed by previous researchers and us could be by lowering the SK activation levels by yet-explored mechanisms.

### Effect of the Single-Nucleotide Polymorphism (A400T) in PhoR on Intracellular Growth of Complemented *Mycobacterium tuberculosis* H37Ra Strain in Host Cells

**FIGURE 6:**
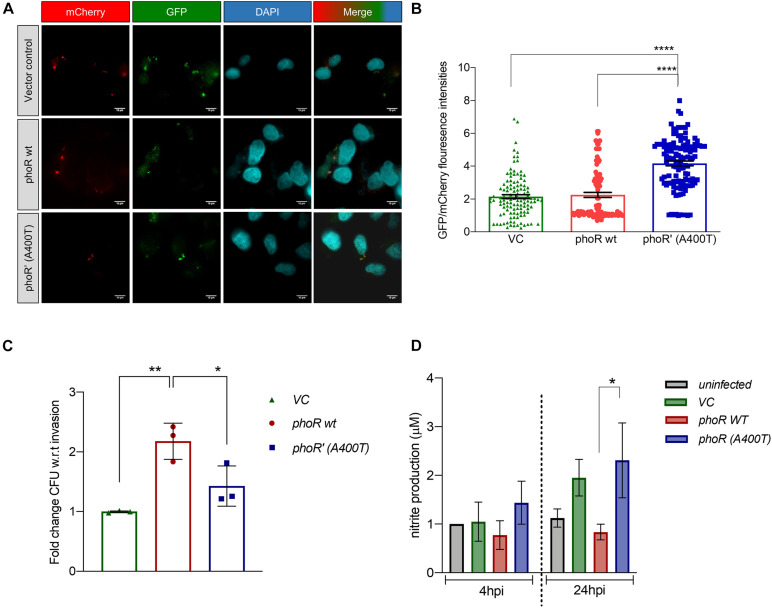
Analysis of the effect of the *phoR* polymorphism by infection studies in host cells using H37Ra strain as a host strain. **(A)** Fluorescence microscopy images showing *aprA* induction upon infection in H460 lung epithelial cell line at 4 hpi. Top panel, a strain carrying vector alone; middle panel, PhoR wt; and lower panel, PhoR’ (A400T) mutant. mCherry marks constitutive promoter activity; green fluorescent protein (GFP), *aprA* marks promoter activity; DAPI marks the nucleus of the host cell; and merged image shows localization for all fluorophores. The brightness of merged image was increased to show presence of bacillary signal. **(B)** Quantification of relative fluorescence 4 hpi of intracellular bacilli by GFP/mCherry ratio for ∼100 bacilli. Cells were infected with *H37Ra* strains carrying *aprA* promoter-reporter plasmid along with an integrated copy of the empty vector, or vector containing either the wild-type or mutant *phoPR* operon. **(C)** Quantitation of the intracellular bacterial burden for various strains (as indicated) after infection in THP-1 differential macrophage cell line. Cells were infected with *H37Ra* strains an integrated copy of the empty vector, the wild-type or mutant *phoPR* operon. Colony-forming units (CFUs) obtained at 24 hpi are shown. **(D)** Measurement of NO production in supernatants of uninfected and infected THP-1 cells by the Griess test at various time points. Cells were infected with strains described above, and the NO/nitrite levels at 4 hpi were measured; left panel, 24 hpi; right panel, 72 hpi. The nitrite produced is normalized to the uninfected cells at 4 hpi. For all experiments, *n* = 3, where n represents the number of biological replicates used in the experiments. *p*-values, * ≤ 0.05, ** ≤ 0.01, *** ≤ 0.001, and **** ≤ 0.0001, were determined by one-way ANOVA.

Having established that the SNP alters expression of PhoP-regulated genes *in vivo*, by quantifying the *aprA* reporter activity at various time points post-infection (4 hpi), we found that the expression of *aprA*-GFP was persistently higher in the mutant strain, and hence quantified the promoter activity for ∼100 bacilli of each strain inside the infected H460 lung epithelial cell line (Figures 6A,B). We also performed these experiments in the presence of the inhibitor ETZ at a concentration of 100 μM. This concentration does not affect the host cells’ health or the bacteria (Supplementary Figure 4B). We recorded differences in the expression of *aprA*-GFP, with the mutant showing higher expression of *aprA*-GFP at any given time point (Figure 6B). We also investigated the ability of the mutant strain to survive within differentiated THP-1 cells, with the three test strains of H37Ra carrying the pCV125 empty vector, the *pho R* WT, or *phoR*’ (A400T) at an MOI of 10 and plated for colony-forming unit (CFU) differences at 24 hpi and normalized to the invasion time point at 4 hpi (Figure 6C). Interestingly, while the complementation with the WT PhoR increased the CFU by more than twofold, the presence of mutant PhoR only showed marginal increase in the bacterial load, suggesting an impaired survival of the strain, similar to vector alone (Figure 6C). Thus, we hypothesize that inefficient survival within cells may be a key factor for this SNP not being selected naturally in the population.

The survival advantage of the strain carrying the WT PhoPR protein could plausibly be driven by alteration in host responses such as cytokine levels and the oxidative burst (Voskuil et al., 2011). We examined if the host responds differently to the WT and mutant strains, by monitoring the NO levels from infected THP-1 cells at various time points (4 and 24 hpi) using the Griess reagent. In agreement with previously published data (41), the NO levels in the cells infected with the WT PhoR strain were always lower owing to an active *phoPR* regulon of H37Rv (Figure 6D; Ferrer et al., 2010), which is reflected in the higher CFU load obtained from them. Interestingly, the production of NO when infected with the strain containing mutant PhoR’ (A400T) was ∼1.5-fold higher than the WT and similar to the strain containing the empty vector and at 24 hpi (Figure 6D, right panel). This response was captured on the bacterial burden as well (Figure 6C), and both vector control and mutant PhoR strains had similar CFU, which was lower than that of WT PhoR. Thus, the host response could be a significant contributor to the absence of selection of this mutation in clinical strains, as the increase in NO could be one of the ways the host eradicates the mutant strain more effectively. These observations reveal that multiple factors drive infection and survival in the host, such as the adaptivity of the strain to infect and survive in the host and highlight the finding that the PhoR polymorphism at A400T position, which though robustly activates the *phoPR* TCS and downstream operon activity, is not favorable for invasion and survival conditions *in vivo*.

Overall, we present experimental evidences underlying the occurrence of a poorly prevalent polymorphism of PhoR SK in clinical strains, which improves its biochemical activities but reduces its overall fitness by affecting its ability to invade and survive in the intracellular environment *in vitro*.

## Discussion

Various reports on genetic variations among strains of *M. tuberculosis* used in the laboratory and clinical strains address the diversity that exists among them (Ramaswamy and Musser, 1998; Fleischmann et al., 2002; Zheng et al., 2008; Schreuder et al., 2015). However, these reports seldom address the variations seen in the TCS regions of clinical strains nor addresses their effects on signaling. We report and characterize a rare polymorphism in the PhoR SK protein that establishes an altered signaling landscape in the tubercle bacteria.

The PhoPR TCS is relatively well-studied in *M. tuberculosis* and implicated in regulating many essential processes such as virulence, immunogenicity, persistence, lipid synthesis and metabolism, cell wall composition, and aerobic and anaerobic respiration (Pérez et al., 2001; Gupta et al., 2006; Walters et al., 2006; Gonzalo-Asensio et al., 2008; Pathak et al., 2010). The PhoR histidine SK protein has an integral membrane-anchored domain, with an extracellular domain to sense extracellular cues (Ryndak et al., 2008) and a typical kinase domain containing the SHELR motif with the conserved histidine residue as phosphorylation site. The mutation A400T, which maps in the HATPase domain of the protein, enhances its catalytic activity in terms of stable phosphotransfer and reduced autophosphatase activity, which is rarely examined, ultimately affecting the quantum of the activated RR PhoP available in the system. The mutation abrogated the rapid decay of phosphorylated SK and RR, which we generally record for the PhoPR system, thereby facilitating more persistent activation of the PhoPR regulon. This change enhanced the levels of PhoP∼P, leading to enhancement in its DNA binding to target gene promoters, such as for the *aprA* (acid and phagosome regulated) locus. These changes when tested *in vivo* in acidic pH revealed robust activation of the H^+^ sensing TCS, PhoPR in the mutant strain.

For our studies, we used an avirulent mycobacterial strain, H37Ra, which has a mutation that impairs the DNA-binding activity of the PhoP protein (Chesne-Seck et al., 2008; Zheng et al., 2008). The strain offered us an advantage, as the mutation’s effect can be recorded without generating a knockout strain. The impact of the SK protein’s altered activity in the A400T mutant is still profoundly seen at the levels of downstream genes, highlighting the utility of H37Ra in such studies.

Besides *aprA*, expression analysis of the *phoR*, *phoP*, and *pks2*, other downstream genes, showed higher levels in the strains carrying WT or mutant *phoR* gene, unlike one carrying the vector only. When the expression was analyzed by growing the cultures at a pH of 5.5 over 12 days, an induction was recorded for all of them, proving that the complementation of *phoPR* into H37Ra reconstitutes the functional PhoPR operon; however, there was no induction of another TCS gene *tcrX* when tested. However, a significantly higher expression was observed for the mutant PhoR strain due to its enhanced kinase activity and impaired phosphatase activity. In the functional complementation background, the readout from *aprA* promoter-reporter plasmid (Abramovitch et al., 2011) showed a higher expression in the presence of mutant PhoR compared with the WT PhoR and the vector control, thus indirectly providing evidence that low pH is linked to activation of PhoR, enhancing the kinase activity of the PhoR protein. This aspect has not been established to date owing to the lack of biochemically characterized full-length proteins.

Based on the experimental evidence, it was tempting to hypothesize that this improved response to acidic pH could benefit the mutant strain. However, in infection studies, we recorded lower bacterial burden in the mutant strain, compared with the strain carrying WT PhoPR locus. This observation correlated well with the changes in the NO levels in the infected cells, where the WT showed reduced NO levels and higher bacterial burden.

Our results display the importance and selection of a regulated and evolutionarily tuned signaling cascade during infection. Even though the polymorphism seems to provide a functional advantage and primes the *phoPR* operon for environmental stimuli, early and higher expression of the target genes is not favorable for the bacteria during infection. Similar observations have been made for several viral infections and mutations with a low invasion profile and are not very successful. For instance, HIV-1 subtype C accounts for over 50% of the conditions globally. However, it is less fit within-host than the less prevalent HIV-1 subtype B (Shet et al., 2016), possibly because of its relatively improved ability to be transmitted successfully to new hosts (Ariën et al., 2007).

An exciting offshoot of our study is the implication of SNPs on drug resistance. In an attempt to ensure that PhoPR is the primary regulator of our observations under low pH, we used an inhibitor ETZ, previously reported to inhibit acid sensing in this system. While the inhibitor lowered the expression of target gene *aprA* and reduced CFU in cell line infection, this inhibitor when tested in the autophosphorylation assay showed reduced inhibitory activity against the mutant protein. This observation suggests the importance of characterizing the effect of inhibitors vis-à-vis polymorphisms seen in the target protein in clinical strains, which would affect the efficacy of various new therapeutic entities.

**FIGURE 7:**
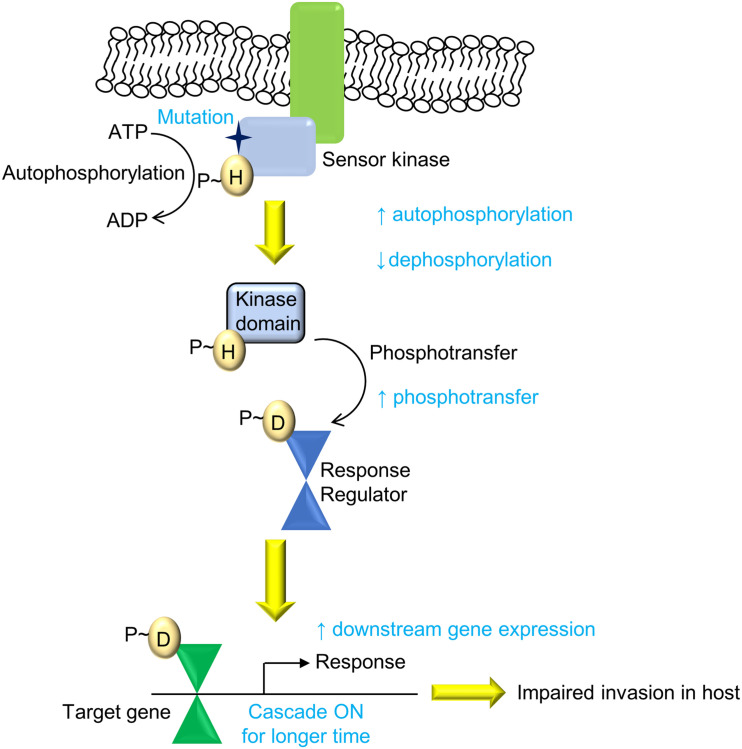
Summary of the PhoPR two-component system (TCS) signaling cascade and the effect of the A400T mutation. The mutation (A400T) in the sensor kinase (SK) PhoR, which responds to low pH, enhances downstream gene expression because of higher autophosphorylation and reduced autophosphatase activity. This leads to enhanced phosphorylation of PhoP protein and its DNA-binding ability (shown in red). The signaling changes affect the invasion of the bacteria that harbor the mutation and reduce the prevalence of the strain carrying single-nucleotide polymorphism (SNP) in the population.

Overall, we present an evolutionary design of signaling systems in bacteria, wherein genetic polymorphisms that can bring about an increase in signaling may not be favorable for the bacteria and hence may be deselected. Here, the mutation in the SK PhoR had better activity but limited invasion ability, possibly through altered host responses upon infection (Figure 7), facilitating its clearance through processes such as the production NO, which limits its replication within the host. Though the mechanisms by which this is governed are yet to be elucidated, such changes confer increased pathogenesis but impaired transmission in clinical strains such as in *M. tuberculosis* IS*6110* B strain (Soto et al., 2004; Broset et al., 2015). Our finding supports the understanding that the balance between gene expression levels and their temporal dynamics needs to be optimized to maintain pathogenicity and virulence.

## Data Availability Statement

The original contributions presented in the study are included in the article/Supplementary Material, further inquiries can be directed to the corresponding author/s.

## Author Contributions

UW designed the study, performed the experiments, analyzed the data, and wrote the manuscript. MK performed project administration and supervision. VM analyzed the data and wrote the manuscript. ND provided conceptualization and project administration. DS conceived the study, analyzed the data, and wrote the manuscript. All authors contributed to the article and approved the submitted version.

## Conflict of Interest

The authors declare that the research was conducted in the absence of any commercial or financial relationships that could be construed as a potential conflict of interest.

## Publisher’s Note

All claims expressed in this article are solely those of the authors and do not necessarily represent those of their affiliated organizations, or those of the publisher, the editors and the reviewers. Any product that may be evaluated in this article, or claim that may be made by its manufacturer, is not guaranteed or endorsed by the publisher.

## Footnotes

1
https://mtb.dobzhanskycenter.org
