# Supplementary material for: A Low-Prevalence Single-Nucleotide Polymorphism in the Sensor Kinase PhoR in Mycobacterium tuberculosis Suppresses Its Autophosphatase Activity and Reduces Pathogenic Fitness: Implications in Evolutionary Selection
Source: Front Microbiol. 2021 Aug 25;12:724482. doi: 10.3389/fmicb.2021.724482 (PMC8424205; doi:10.3389/fmicb.2021.724482)
Supplement: Supplementary file 1 [file Data_Sheet_1.PDF]

SUPPLEMENTARY TABLE

1) List of primers used in this study.

Primers	Sequence	Reference
***phoR* A400T fwd**	5′ CCTCGAGGTCACCGATGACGGTCCGG 3′	This study (cloning primer)
***phoR* A400T rvs**	5′ CCGGACCGTCATCGGTGACCTCGAGG 3′	
***phoPR* pCV fwd**	5′ GATCCATATGTCAGGGCGGCCCTGGCACAACTGGC 3′	This study (cloning primer)
***phoPR* pCV rvs**	5′ CCGAAGCTTCCGGTCCATCGGCCCGT 3′	
***aprA* prom fwd**	5′ GCCGCACTGACGCTGATGATCC 3′	(1)
***aprA* prom rvs**	5′ CTCTGTCCCCCTTCCGAGCC 3′	
***pks2* fwd**	5′ GTTGTGGAAGGCGTTGTTAC 3′	(2)
***pks2* rvs**	5′ GTCGTAGAACTCGTCGCAAT 3′	
***phoP rt* fwd**	5′ AGACCCACGAAGTGTGGAAG 3′	(3)
***phoP rt* rvs**	5′ CGTGGTCGAGAATCTTAGGC 3′	
***phoR rt* fwd**	5′ GGACCCCTACCCTGGTATA 3′	(3)
***phoR rt* rvs**	5′ ATGGCAGTGTTGTCGTTGAG 3′	
***16s* rRNA rt fwd**	5′ ACGCGAAGAACCTTACCTGG 3′	(4)
***16s* rRNA rt rvs**	5′ CACCTTCCTCCGAGTTGACC 3′	

2) List of Plasmids used in this study

Plasmids	Source	Reference
**Rv0757, PhoP cloned in pPROEx-HT vector**	Lab collection	(5)
**Rv0758, PhoR cloned in pPROEx-HT vector**	Lab collection	
**pProEx-HT PhoR’ A400T**	Lab collection	This study
**pCV125**	Lab collection	(6)
**pCV125- *phoPR***	Lab collection	This study
**pCV125- *phoPR’* A400T**	Lab collection	This study
aprA-egfp2-mCherry	Gift	(7)

References

1. Bansal R, Anil Kumar V, Sevalkar RR, Singh PR, Sarkar D. Mycobacterium tuberculosis virulence-regulator PhoP interacts with alternative sigma factor SigE during acid-stress response. Mol Microbiol. 2017;104(3):400–11.

2. Goyal R, Das AK, Singh R, Singh PK, Korpole S, Sarkar D. Phosphorylation of PhoP protein plays direct regulatory role in lipid biosynthesis of Mycobacterium tuberculosis. J Biol Chem. 2011;286(52):45197–208.

3. Gonzalo-Asensio J, Soto CY, Arbués A, Sancho J, Menéndez MDC, García MJ, et al. The Mycobacterium tuberculosis phoPR operon is positively autoregulated in the virulent strain H37Rv. J Bacteriol. 2008;190(21):7068–78.

4. Singh KK, Bhardwaj N, Sankhe GD, Udaykumar N, Singh R, Malhotra V, et al. Acetylation of Response Regulator Proteins, TcrX and MtrA in M. tuberculosis Tunes their Phosphotransfer Ability and Modulates Two-Component Signaling Crosstalk. J Mol Biol. 2019 Feb;431(4):777–93.

5. Agrawal R, Pandey A, Rajankar MP, Dixit NM, Saini DK. The two-component signalling networks of Mycobacterium tuberculosis display extensive cross-talk in vitro. Biochem J. 2015;469:121–34.

6. Alland D, Steyn AJ, Weisbrod T, Aldrich K, Jacobs WR. Characterization of the Mycobacterium tuberculosis iniBAC promoter, a promoter that responds to cell wall biosynthesis inhibition. J Bacteriol. 2000;182(7):1802–11.

7. Abramovitch RB, Rohde KH, Hsu FF, Russell DG. aprABC: A Mycobacterium tuberculosis complex-specific locus that modulates pH-driven adaptation to the macrophage phagosome. Mol Microbiol. 2011;80(3):678–94.
