# Supplementary material for: A Low-Prevalence Single-Nucleotide Polymorphism in the Sensor Kinase PhoR in Mycobacterium tuberculosis Suppresses Its Autophosphatase Activity and Reduces Pathogenic Fitness: Implications in Evolutionary Selection
Source: Front Microbiol. 2021 Aug 25;12:724482. doi: 10.3389/fmicb.2021.724482 (PMC8424205; doi:10.3389/fmicb.2021.724482)
Supplement: Supplementary file 2 [file Data_Sheet_2.PDF]

S1 Fig

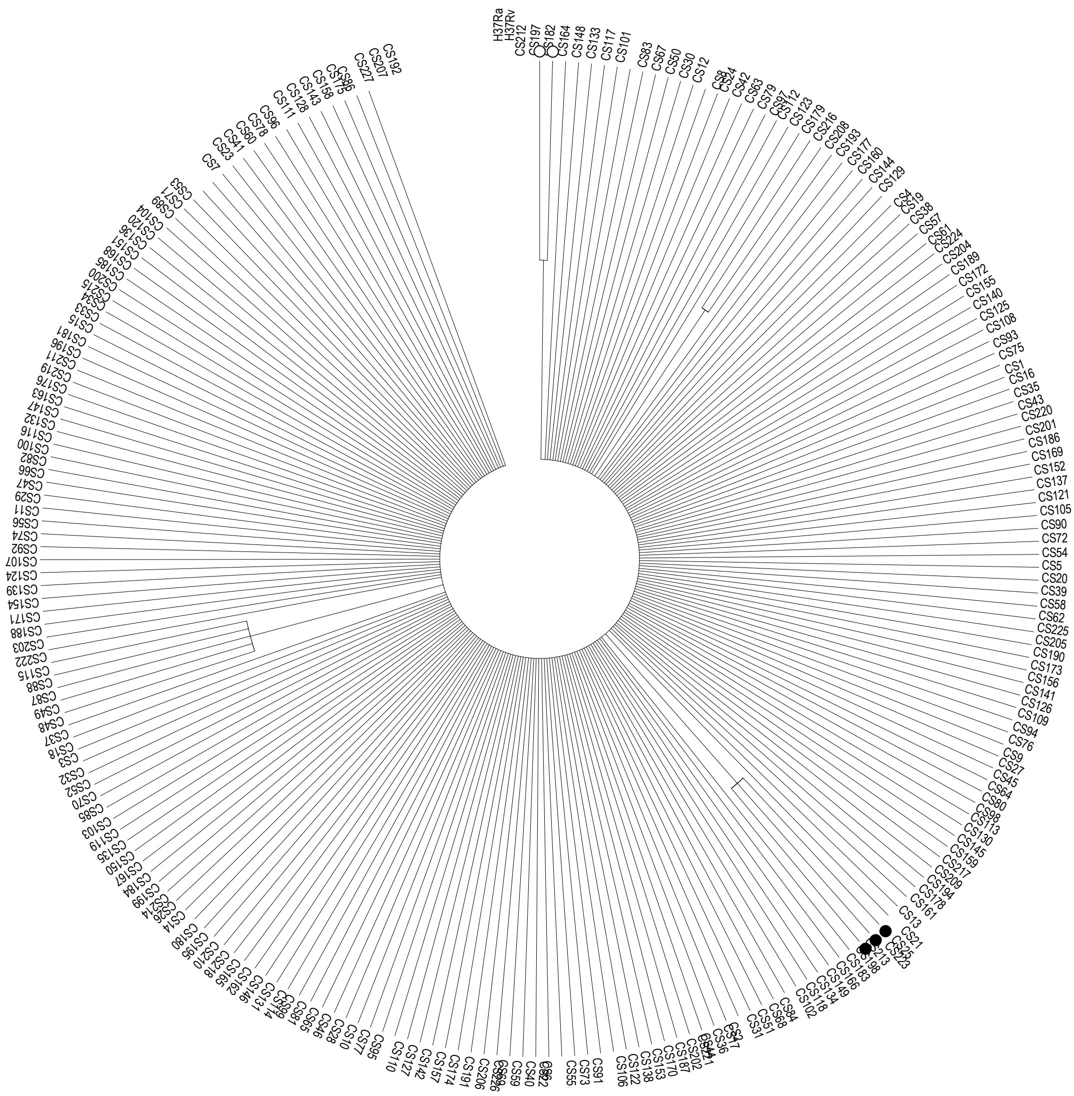

Figure S1. Radial phylogenetic tree of PhoR homologs from *M. tuberculosis H37Rv, H37Ra,* and the 223 clinical strains analyzed in this study. The map was generated by aligning sequences for the *phoR* gene. The phylogenetic tree was build using the Maximum-likelihood method based on the Tamura-Nei model by MEGA. Bootstrap analysis was performed with 500 replicates (1). The three strains which carry the A400T mutation is marked with black dots.

S2 Fig

A.

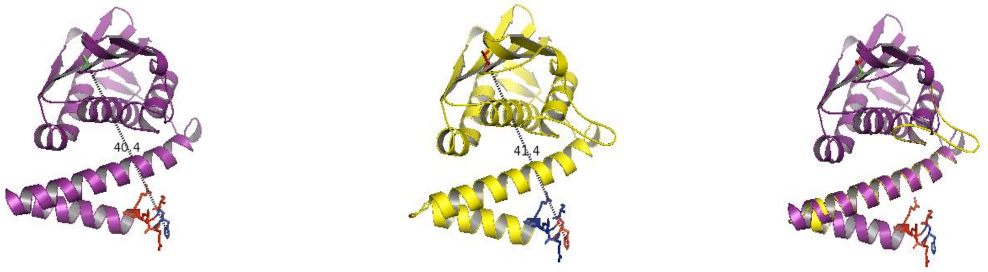

PhoR wt | PhoR’ **A400T** in the HATPase domain | Overlay of wt and mutant protein structures

(i) **(ii)** **(iii)**

B.

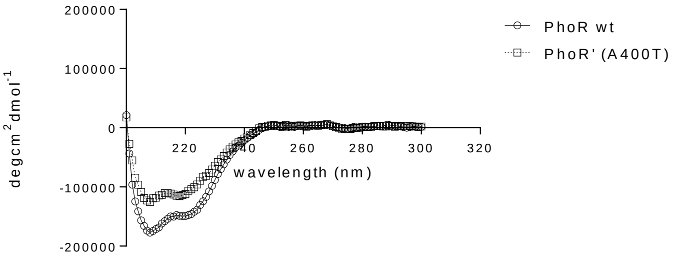

Calculated by k2d3

**Protein**	**α helix**	**β strand**
PhoR wt	37.42%	15.28%
PhoR’ (A400T)	37.78%	15.33%

Figure S2. A. Predicted ribbon structure models of the sensor kinase protein PhoR, using PHYRE (2) for (i) wildtype PhoR protein (PhoR wt) (ii) mutant PhoR protein (PhoR’ A400T) (iii) Overlay, showing the kinase and HATPase domain only. The distance between the conserved Histidine residue and the Alanine residue (in the wild type protein)/ Threonine residue (in the mutant protein) was measured and marked. The overlay marks shows no gross changes in the secondary structure between the wild type and mutant proteins. **B.** Circular dichroism analysis of PhoR wt and PhoR’ (A400T) proteins using a Jasco Spectropolarimeter. Ellipticity was measured from 200 nm to 300 nm at 50nm/ sec scanning speed and 1 mm pathlength. The data were analysed with K2D3 Dichroweb software.

S3 Fig

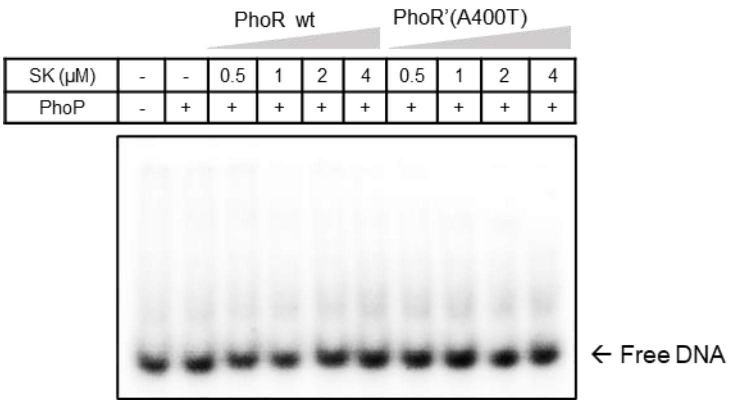

S3 Fig. Electrophoretic mobility shift assay (EMSA) for PhoP binding to with *aprA* promoter region. A dose titration on DNA binding activity of PhoP in the presence of PhoR wt and PhoR' (A400T) proteins. A 500 bp DNA fragment corresponding to the promoter region of the *aprA* gene was PCR amplified from *M. tuberculosis* H37Rv genomic DNA using specific primers. EMSA was performed using PhoP (1μM) and increasing PhoR wild-type and mutant proteins concentrations as indicated. The concentration gradient indicates the amount of PhoR wt and PhoR' (A400T) used in the assay.

S4 Fig

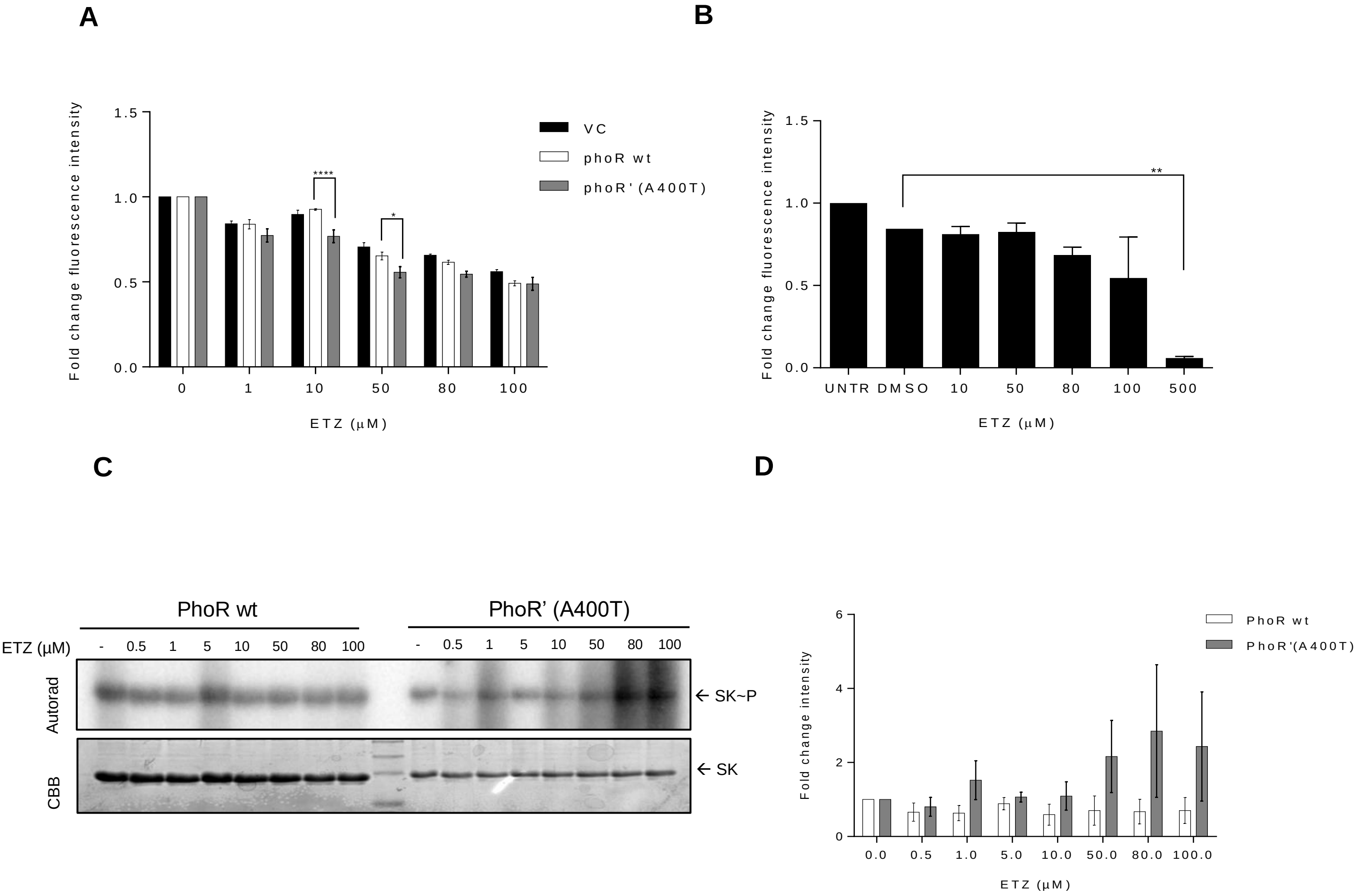

S4 Fig. A. Alamar blue assay after 12 days of treatment with various ETZ concentrations on *M. tuberculosis* H37Ra strain carrying vector alone or wt or mutated PhoPR TCS. The fluorescence read at 560 nm excitation and 590 nm emission was normalized to vehicle control DMSO for each strain. **B.** Resazurin assay to measure ETZ's toxicity on mammalian cells, 3 days post-treatment in the presence of various concentrations as indicated. The fluorescence intensities were normalized to that of the untreated cells. **C.** Autophosphorylation analysis of the PhoR wt and PhoR' (A400T) proteins in the presence of increasing concentrations of ETZ at 2 hours. **D.** Quantification of the SK~P formed in the presence of the inhibitor ETZ at different concentrations. n=3 for each experiment. For all experiments n=3 and p values; * ≤ 0.05, ** ≤ 0.01, *** ≤ 0.001, **** ≤ 0.0001 were determined by two-way ANOVA.

S5 Fig

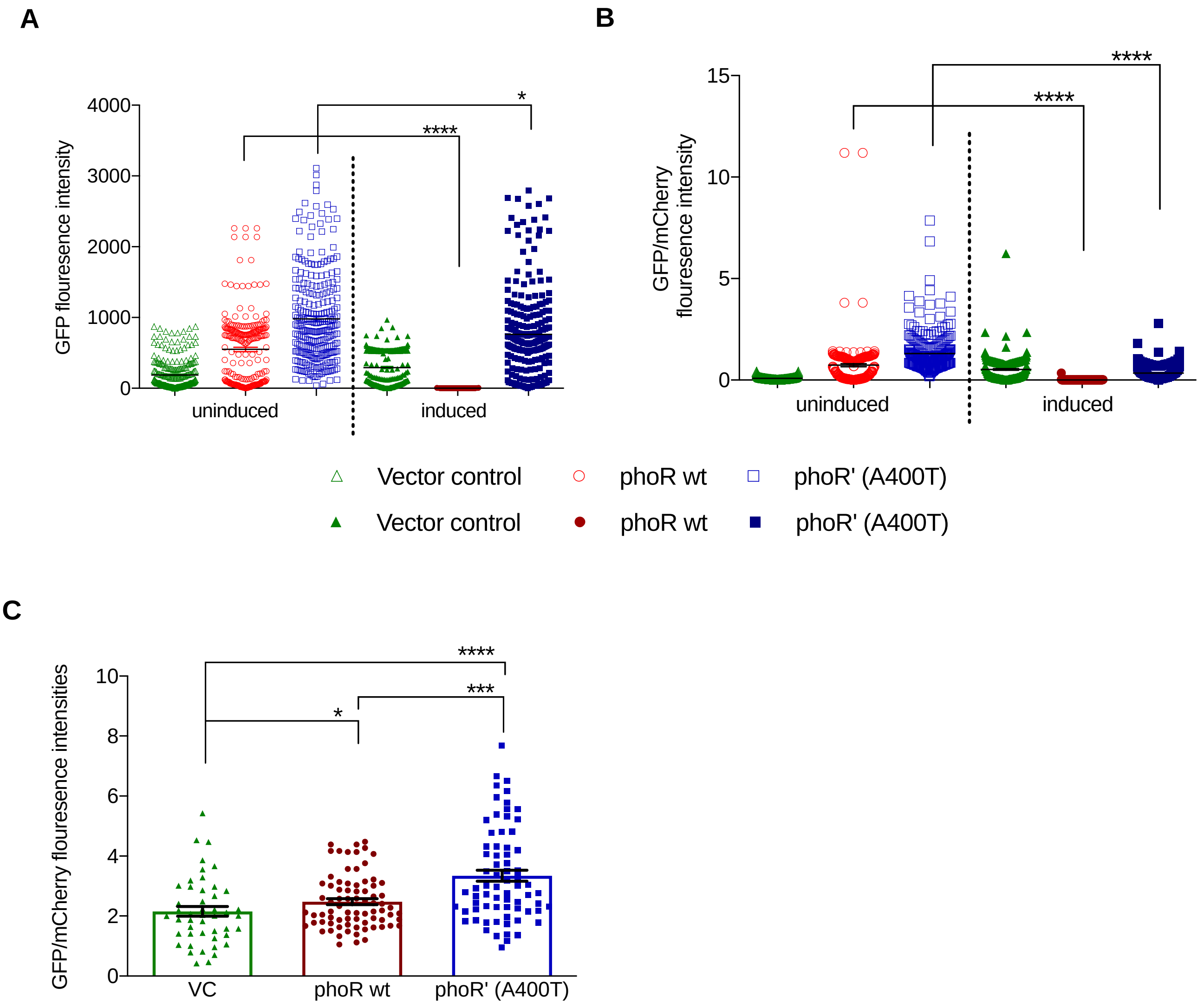

S5 Fig. A. Quantitation of PhoP transcriptional response by measuring GFP fluorescence in H37Ra carrying vector alone or WT or mutated PhoPR TCS. The cultures were exposed to low pH (5.2) for induction in the presence of the inhibitor ETZ at day 12 post-induction. The fluorescence data were collected from ~300 individual bacilli, using flouresence microscopy. **B.** The data in A represented as a ratio of GFP/mCherry. **C.** Quantification of relative fluorescence at 4hpi in intracellular bacilli described as GFP/mCherry ratio from ~300 bacilli in infected cells in the presence of the inhibitor ETZ (80µM). For all experiments n=3, p values; *≤ 0.05, **≤ 0.01, ***≤ 0.001, ****≤ 0.0001 were determined by two-way ANOVA.

References.

1. Felsenstein J. Phylogenies and the Comparative Method Author (s): Joseph Felsenstein Source : The American Naturalist , Vol . 125 , No . 1 (Jan ., 1985), pp . 1-15 Published by : The University of Chicago Press for The American Society of Naturalists Stable URL : ht. 1985;125(1):1–15.

2. Kelley LA, Sternberg MJE. Protein structure prediction on the web: A case study using the phyre server. Nat Protoc. 2009;4(3):363–73.
